# Channel-engineered GAA FETs: From device to circuit perspective for Angstrom technology nodes

**DOI:** 10.1038/s41598-026-58015-x

**Published:** 2026-06-19

**Authors:** Vakkalakula Bharath Sreenivasulu, Narasimhulu Thoti

**Affiliations:** 1 Manipal Institute of Technology Bengaluru, Manipal Academy of Higher Education, Manipal, India; 2 Microelectronics Research Unit, Faculty of Information Technology and Electrical Engineering, University of Oulu, Oulu, Finland

**Keywords:** C-FET, GAA, I-FET, Inverter, Nanosheet, Ring oscillator, Engineering, Materials science, Nanoscience and technology, Physics

## Abstract

This work presents a comprehensive device-to-circuit analysis of channel-engineered gate-all-around (GAA) FETs intended for advanced CMOS logic applications, namely the comb-shaped FET (C-FET) and inter-bridge FET (I-FET), benchmarked against a conventional nanosheet FET (NS-FET). All devices are designed according to sub-3-nm IRDS guidelines and evaluated through calibrated 3D TCAD simulations. The results show that the incorporation of vertical inter-bridge (IB) channels in C-FET and I-FET significantly enhances the effective width, leading to notable improvements in drive current, with the I-FET achieving the highest $I_\textrm{ON}$ and $I_\textrm{ON}/I_\textrm{OFF}$ ratio. Scalability studies reveal that the I-FET retains superior performance under variations in gate length and IB dimensions while maintaining acceptable short-channel behavior. Analog and RF evaluations indicate that both C-FET and I-FET exhibit enhanced transconductance, cut-off frequency, and reduced intrinsic delay compared to NS-FETs, making them promising candidates for high-speed applications. Circuit-level simulations using LUT-based Verilog-A models further confirm that C-FET and I-FET deliver higher switching currents and improved ring-oscillator frequencies, with the I-FET achieving the maximum $f_\textrm{OSC}$ across all supply voltages and stage counts. Overall, the results demonstrate that channel-engineered C-FET and I-FET architectures provide substantial performance advantages over conventional nanosheet devices, highlighting their suitability for advanced CMOS and RF systems for angstrom technology nodes.

## Introduction

Current fin-field effect transistor (FinFET) technology requires increasingly thinner and taller fins, which lead to severe electrostatic fluctuations and degradation in the drive current ($I_{\textrm{DS}}$). Additionally, sustaining adequate drive strength in highly scaled fin-shaped standard cells remains a major challenge^1–3^. To address these limitations, stacked nanosheet (NS) gate-all-around (GAA) FETs have emerged as the most promising successor to FinFETs due to their superior gate electrostatics, higher current density per footprint, and tunable sheet width^4,5^. However, as the NS layers are vertically stacked more closely, parasitic interactions increase, adversely affecting device and circuit performance^2,3^. Consequently, innovative channel geometries are required to enhance on-current ($I_{\textrm{ON}}$) while minimizing parasitic effects.

Several channel-engineered architectures–such as U-shaped, comb-shaped (C-shaped), and tree-/I-shaped channels–have recently gained interest as potential alternatives to conventional NS and fin-based devices^6–10^. Among these, C-shaped and I-shaped designs are particularly promising due to their fabrication feasibility and strong electrostatic control^6^. The I-shaped FET (I-FET) can be viewed as a hybrid design incorporating both NS and vertical inter-bridge (IB) features^6^. It is experimentally realized by inserting a sacrificial layer between NS channels followed by selective etching to create a vertical IB, effectively acting as a fin-like conduction path. In C-shaped FETs (C-FETs), a sidewall-selective epitaxial process forms the channel, significantly enhancing the effective width-to-footprint ratio ($W_{\textrm{eff}}/FP$). Incorporating IBs in both C-FET and I-FET architectures has been shown to increase $I_{\textrm{ON}}$ by up to 76% when compared with NS-FETs^6^, while also mitigating mechanical deformation issues in stacked nanosheet structures.

Beyond these devices, complementary FET (CFET) technology–which vertically stacks n- and p-type GAA devices–has emerged as a system-level integration approach for extending Moore’s law^11–15^. Although conceptually distinct from channel-engineered C-FETs, CFETs share the same scaling motivation and highlight the importance of 3D device integration. Furthermore, tunneling FETs (TFETs) have been explored for ultra-low-power applications due to their sub-60 mV/decade switching capability; however, their limited $I_{\textrm{ON}}$ often restricts their use in high-performance logic^16–18^. These comparisons underscore the need for improved channel structures, such as C-FET and I-FET, that can simultaneously offer high drive strength and favorable electrostatics. In addition, junctionless FET (JLFET) architectures have been investigated for their simplified doping profiles and improved electrostatic control at nanoscale dimensions^19–23^. While such approaches offer advantages in terms of reduced variability and fabrication complexity, their performance trade-offs highlight the continued need for channel-engineered GAA structures for high-performance applications.

Despite notable advancements, a comprehensive evaluation of these emerging channel-engineered GAA devices from both device-level and circuit-level perspectives remains limited. Prior works predominantly focus on DC characteristics, without systematically investigating scalability, analog/RF metrics, and circuit-level implications–key factors for enabling practical adoption of these architectures in advanced technology nodes.

This work focuses on channel-engineered gate-all-around (GAA) transistor architectures for advanced CMOS logic technologies rather than sensing applications. In particular, we investigate and benchmark three device structures: the conventional nanosheet FET (NS-FET), the comb-shaped or C-shaped FET (C-FET), and the inter-bridge FET (I-FET). Although prior studies have reported individual device-level characteristics of these architectures, a comprehensive comparison that connects device physics to circuit-level implications is still lacking.

In this paper, we present a systematic device-to-circuit evaluation of NS-FET, C-FET, and I-FET structures designed according to IRDS projections for sub-3 nm technology nodes. The analysis includes (i) DC performance benchmarking, (ii) scalability assessment with respect to gate length and inter-bridge geometry, (iii) analog and RF performance metrics, (iv) circuit-level validation through CMOS inverter and multi-stage ring oscillator simulations using LUT-based Verilog-A models in the Cadence platform. This unified device-to-circuit framework provides deeper insight into the practical advantages of channel-engineered GAA architectures for future high-performance CMOS technologies.

## Device design and simulation setup

**Fig. 1:**
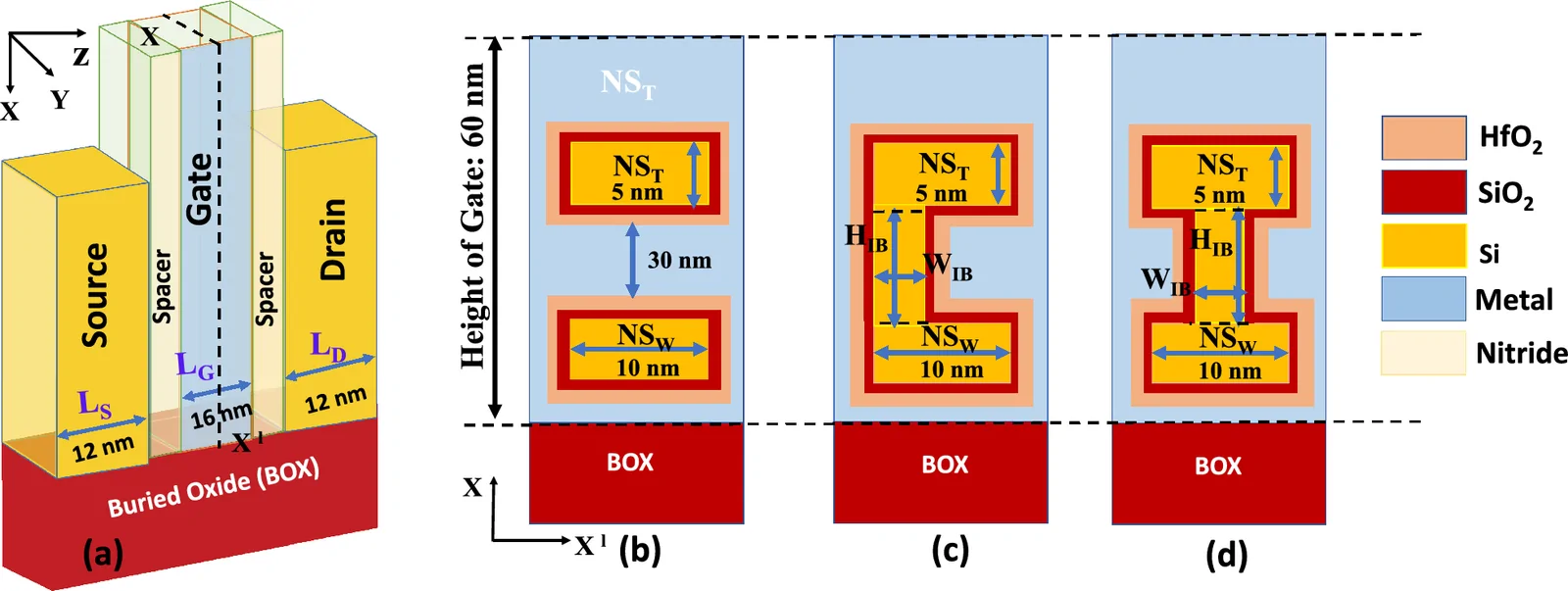
3D and cross-sectional views of (**a**) NS-FET, (**b**) C-FET, and (**c**) I-FET devices are illustrated. Key specifications highlighting the effective width ($\mathrm {W_{eff}}$) are indicated.

The structural layouts of the NS-FET, C-FET, and I-FET devices evaluated in this work are shown in Fig. 1. Key device parameters–summarized in Table 1–follow IRDS guidelines for sub-3 nm nodes, ensuring realistic benchmarking. To maintain a fair comparison across all device architectures, the OFF-state current ($I_{\textrm{OFF}}$) is fixed to the same target value. A dual-layer gate stack composed of SiO$_2$ (0.5 nm) and HfO$_2$ (1.5 nm), corresponding to an effective oxide thickness (EOT) of 0.78 nm, is used to suppress gate tunneling leakage. All devices are implemented on a silicon-on-insulator (SOI) substrate with a buried oxide (BOX) layer, as depicted in Fig. 1, which effectively minimizes substrate parasitic conduction. Nitride spacers are employed along both gate sidewalls to further reduce fringe-induced gate leakage^10^.

**Table 1:** Specifications of proposed device designs.

Device parameter	NS-FET	C-FET	I-FET
Gate length ($L_G$)	16 nm	16 nm	16 nm
Nanosheet thickness ($NS_T$)	5 nm	5 nm	5 nm
Nanosheet width ($N_W$)	10 nm	10 nm	10 nm
Width of inter-bridge ($W_{IB}$)	–	5 nm	5 nm
Height of inter-bridge ($H_{IB}$)	–	30 nm	30 nm
EOT	0.78 nm	0.78 nm	0.78 nm
Spacer length	8 nm	8 nm	8 nm
Source/drain doping (cm$^{-3}$)	$1\times 10^{20}$	$1\times 10^{20}$	$1\times 10^{20}$
Channel doping (cm$^{-3}$)	$1\times 10^{15}$	$1\times 10^{15}$	$1\times 10^{15}$
Gate work function (eV)	4.346	4.367	4.3765

**Fig. 2:**
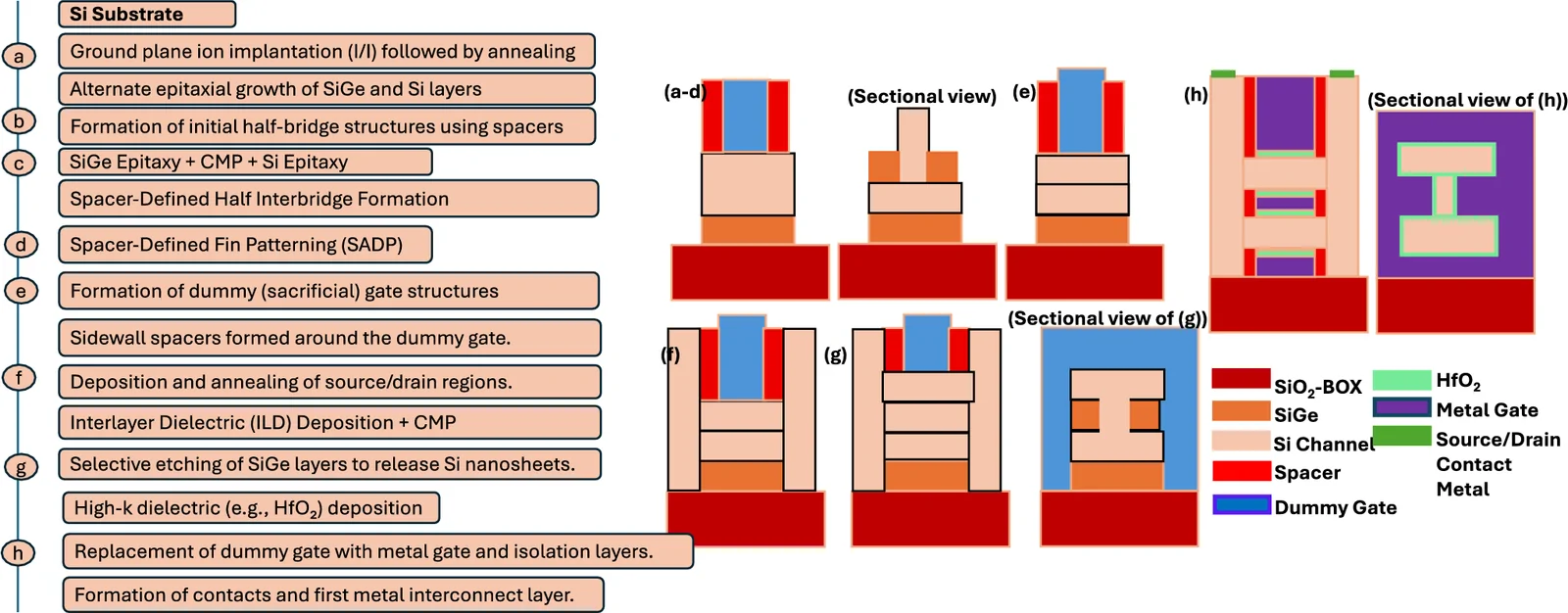
(**a**–**h**) illustrate the simplified fabrication process flow of the I-FET device, highlighting key steps including Si/SiGe multilayer growth, spacer-defined inter-bridge (IB) formation, nanosheet release, and final gate stack and contact formation^9,10^.

**Fig. 3:**
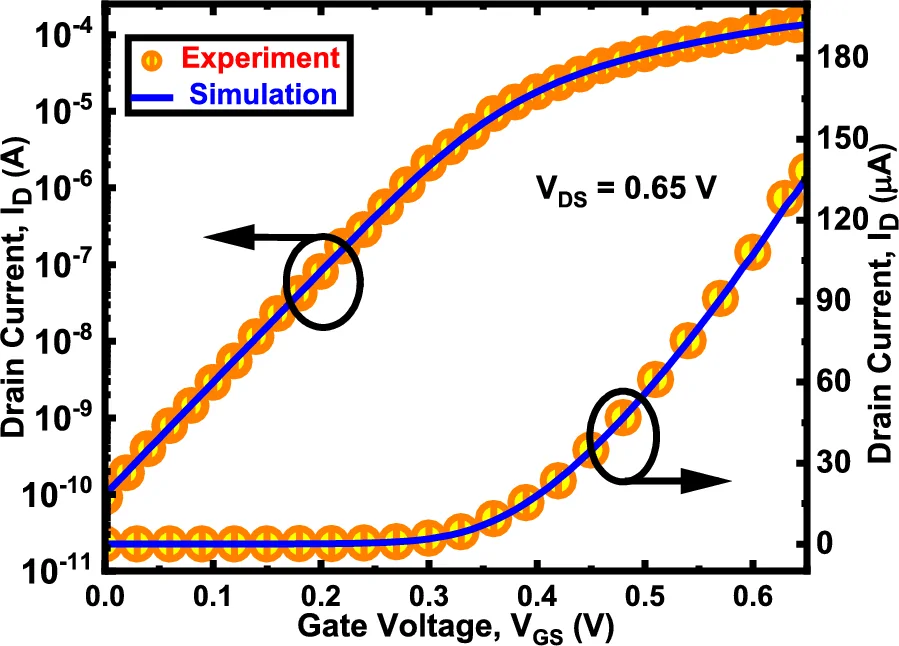
Calibration of the TCAD simulation framework against experimentally reported GAA nanosheet FET data from^5^. The reference device consists of horizontally stacked nanosheet nFET/pFET structures with three nanosheets, inner spacers, gate length $L_G \approx 12$ nm, silicon thickness $T_{Si} \approx 5$ nm, and contacted poly pitch (CPP) of 44/48 nm. The simulated $I_{\textrm{DS}}$–$V_{\textrm{GS}}$ characteristics closely match the experimental results, validating the adopted physical models and calibration methodology.

The fabrication process flow of the I-FET, shown in Fig. 2, illustrates the sequential formation of stacked nanosheet channels along with the vertical inter-bridge (IB) structure. The process begins with substrate preparation and ground-plane formation (a), followed by alternating epitaxial growth of Si/SiGe multilayers to define the stacked channel structure (b). The initial IB regions are defined using spacer-based patterning techniques (c), followed by additional epitaxial growth and planarization steps to complete the stacked structure (d). Subsequently, spacer-defined fin patterning is carried out using self-aligned double patterning (SADP) (e), which further refines the channel geometry. Dummy gate formation and sidewall spacer definition are then performed (f), followed by source/drain formation and interlayer dielectric deposition. Selective etching of the SiGe layers releases the suspended Si nanosheets (g). During this stage, the previously defined spacer regions result in the formation of the vertical IB channel connecting adjacent nanosheets. Finally, high-*k* gate dielectric deposition and metal gate replacement are carried out, followed by contact formation and backend interconnect processing (h). This process flow is consistent with experimentally demonstrated approaches for I-FET and C-FET architectures reported in^6,10^.

The physical modeling and simulation of the NS-FET, C-FET, and I-FET were performed using the Cogenda Visual TCAD platform. The simulations incorporate calibrated physical models to accurately capture electrostatics, carrier transport, and recombination mechanisms in nanoscale GAA devices. The device solver self-consistently couples Poisson’s equation with electron and hole continuity equations under the drift–diffusion transport framework. Quantum confinement effects–especially relevant for ultra-thin nanosheet channels–are incorporated through the density–gradient quantum correction model.

Several physical mechanisms were included to ensure realistic behavior: (1) doping-dependent bandgap narrowing via the Slotboom model; (2) field-dependent mobility (low-field, high-field, and velocity saturation components); (3) quasi-ballistic transport considerations to capture nanoscale carrier dynamics; (4) phonon and Coulomb scattering at the Si/SiO$_2$ interface via the Lombardi mobility model; and (5) Shockley–Read–Hall (SRH) recombination for trap-assisted processes. The drift–diffusion framework with quantum corrections provides a reasonable approximation for the studied devices, including scaled gate lengths down to 10 nm, where transport becomes increasingly quasi-ballistic while comparative trends remain valid.

To validate the accuracy of the simulation framework, an experimentally demonstrated GAA nanosheet FET reported in^5^ was reproduced in TCAD. The reference device consists of horizontally stacked nanosheet nFET/pFET structures featuring three nanosheets, inner spacers, a gate length of approximately 12 nm, silicon thickness ($T_{Si}$) of about 5 nm, and a contacted poly pitch (CPP) of 44/48 nm. The calibration was performed by matching the simulated $I_{\textrm{DS}}$–$V_{\textrm{GS}}$ characteristics with the experimentally reported data under comparable bias conditions. In addition, the calibration approach follows a methodology similar to that reported in^5^, where experimental results are supported by TCAD-based analysis. The close agreement between simulated and experimental characteristics (Fig. 3) confirms that the selected physical models and parameter settings accurately capture real device behavior.

**Fig. 4:**
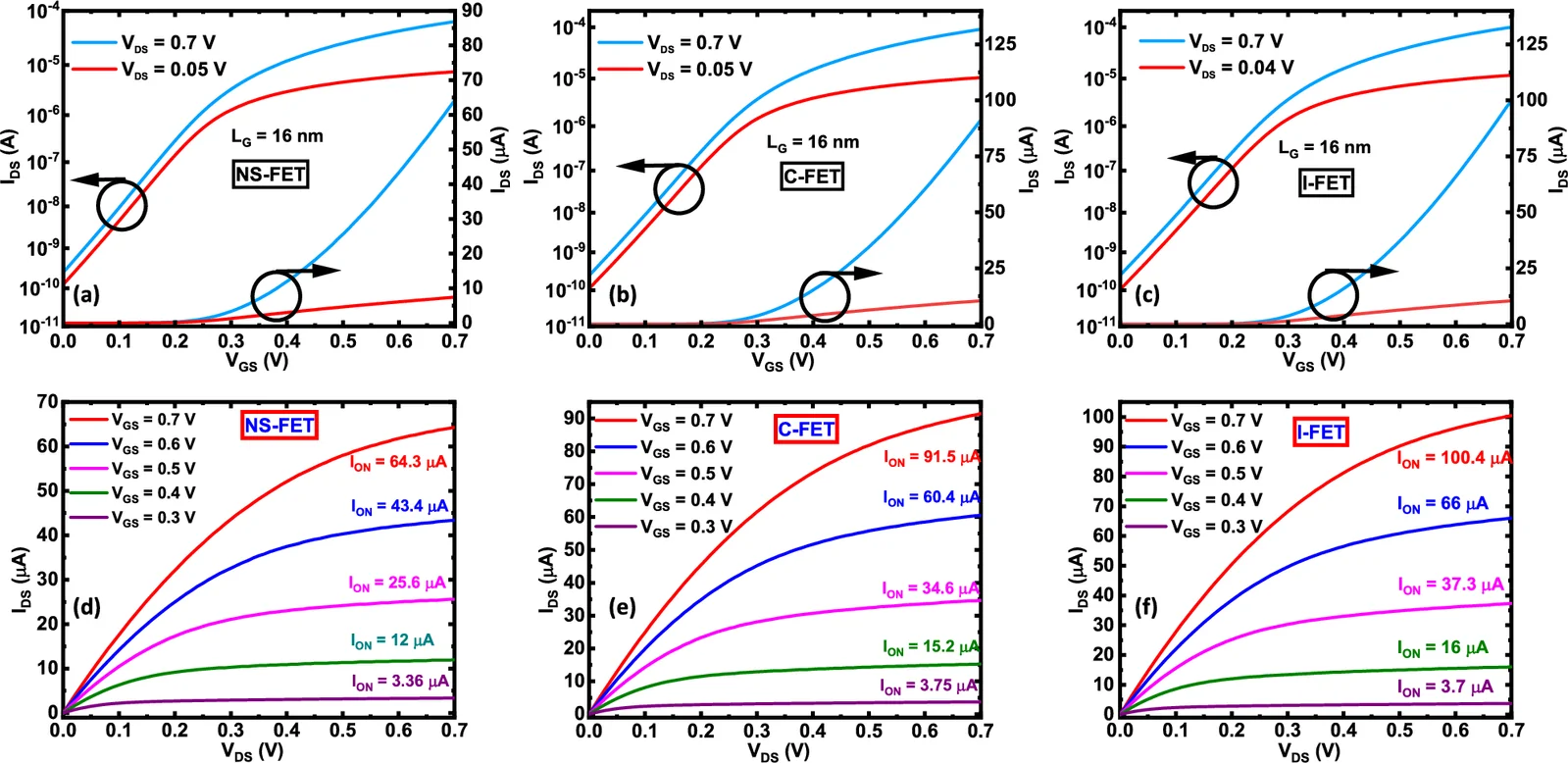
The $\mathrm {I_{DS}}$–$\mathrm {V_{GS}}$ characteristics of (**a**) NS-FET, (**b**) C-FET, and (**c**) I-FET, respectively. Panels (**a**)–(**c**) show device transfer curves at multiple drain biases to highlight threshold, subthreshold slope and on-current behavior, while panels (**d**)–(**f**) illustrate $\mathrm {I_{DS}}$–$\mathrm {V_{DS}}$ for $\mathrm {V_{GS}}$ from 0.3 to 0.7 V, used to extract $\mathrm {I_{ON}}$.

**Fig. 5:**
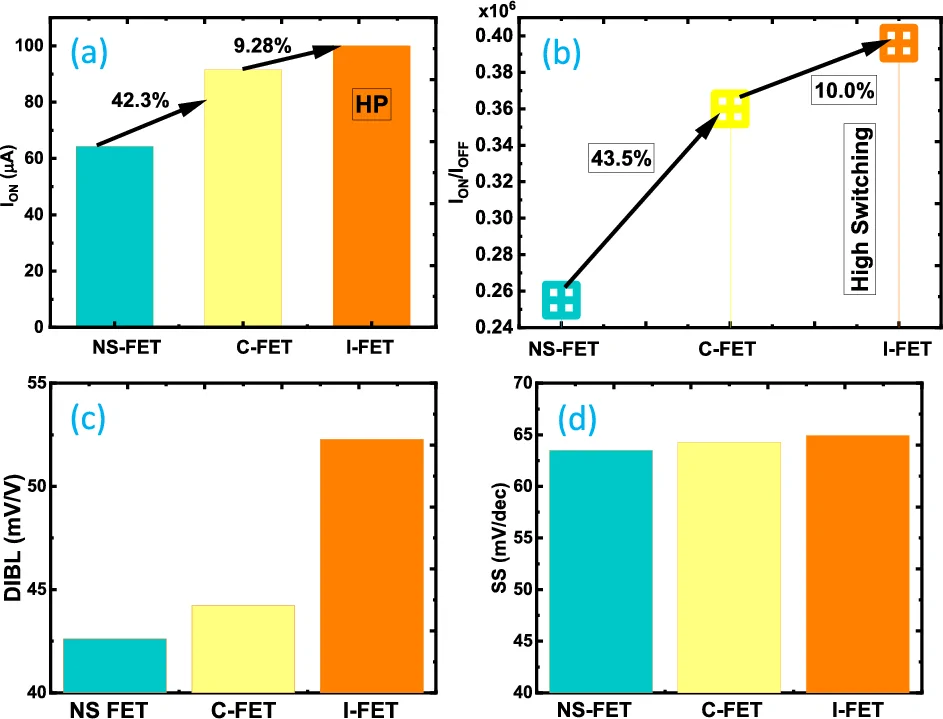
(**a**) $\mathrm {I_{ON}}$, (**b**) $\mathrm {I_{ON}/I_{OFF}}$, (**c**) SS, and (**d**) DIBL of NS-FET, C-FET, and I-FET. The plots summarize device-level trade-offs: I-FET achieves the highest $\mathrm {I_{ON}}$ and $\mathrm {I_{ON}/I_{OFF}}$, while C-FET shows slightly better DIBL/SS in some geometries.

**Fig. 6:**
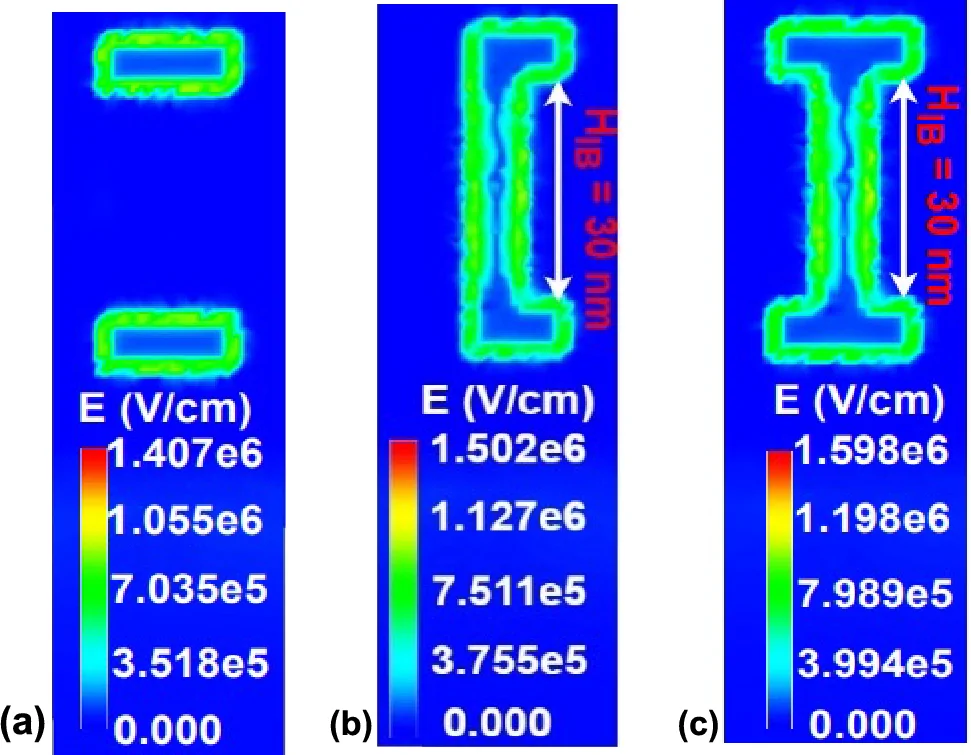
Electric field contour plots along the channel for (**a**) NS-FET, (**b**) C-FET, and (**c**) I-FET at $\mathrm {H_{IB}} = 30$ nm. The I-FET shows stronger and more uniformly distributed channel fields, contributing to its higher drive current.

## Results and discussions

Figure 4a–c shows the transfer characteristics ($I_{\textrm{DS}}$–$V_{\textrm{GS}}$) of NS-FET, C-FET, and I-FET at different drain biases. All devices are compared at a fixed $I_{\textrm{OFF}}$ extracted at $V_{\textrm{DS}}=0.7$ V and $V_{\textrm{GS}}=0$ V to ensure a consistent off-state reference. In this benchmarking approach, the gate work function of each device is tuned to achieve the same $I_{\textrm{OFF}}$, and the ON-state current is then extracted at $V_{GS}=V_{DD}$, which is a commonly adopted methodology for comparing scaled transistor architectures^24–27^. The corresponding output characteristics ($I_{\textrm{DS}}$–$V_{\textrm{DS}}$) for $V_{\textrm{GS}}$ values ranging from 0.3–0.7 V are shown in Fig. 4d–f. From these characteristics, the extracted $I_{\textrm{ON}}$ values at $V_{\textrm{GS}}=0.7$ V are 64.3 *μ*A, 91.5 *μ*A, and 100.4 *μ*A for NS-FET, C-FET, and I-FET, respectively. The higher drive current in C-FET and I-FET arises from their larger effective width ($W_{\textrm{eff}}$) enabled by the C-shaped and inter-bridge assisted geometries, which provide more active channel per footprint than conventional nanosheets.

The key device parameters are extracted using standard methodologies. The threshold voltage ($V_{\textrm{th}}$) is determined using the constant-current method from the transfer characteristics. The subthreshold swing (SS) is extracted from the inverse slope of the $\log (I_{\textrm{DS}})$–$V_{\textrm{GS}}$ curve in the subthreshold region. The drain-induced barrier lowering (DIBL) is calculated as the shift in $V_{\textrm{th}}$ between low and high drain bias conditions. These extraction methods are consistent with established approaches reported in the literature^28^. Figure 5 summarizes the main DC performance metrics, including $I_{\textrm{ON}}$, $I_{\textrm{ON}}/I_{\textrm{OFF}}$, SS, and DIBL. For a consistent benchmarking across device architectures, the gate work function was adjusted such that the $I_{\textrm{OFF}}$ is maintained at the same reference level for all devices. C-FET achieves a 42.3% improvement in $I_{\textrm{ON}}$ over NS-FET, while I-FET provides an additional 9.28% enhancement beyond C-FET. This indicates that I-FET offers the highest performance among the evaluated device architectures, making it highly attractive for high-performance logic. Owing to its increased $W_{\textrm{eff}}$, the I-FET also exhibits the best $I_{\textrm{ON}}/I_{\textrm{OFF}}$ ratio (Fig. 5b), indicating suitability for high-speed digital and DRAM applications^7^.

The DIBL and SS characteristics in Fig. 5c–d show that both C-FET and I-FET experience a slight degradation compared to NS-FET. This behavior originates from the inter-bridge region, where reduced gate coverage and additional channel intersections introduce sub-fin leakage and weaker electrostatics. Nonetheless, the extracted SS and DIBL values remain within acceptable limits for future IRDS-defined technology nodes at $V_{\textrm{DS}}=0.7$ V. Thus, although the introduction of IB structures yields a minor compromise in subthreshold behavior, the significant gains in drive current justify further refinement of IB geometry to balance electrostatics and performance.

**Fig. 7:**
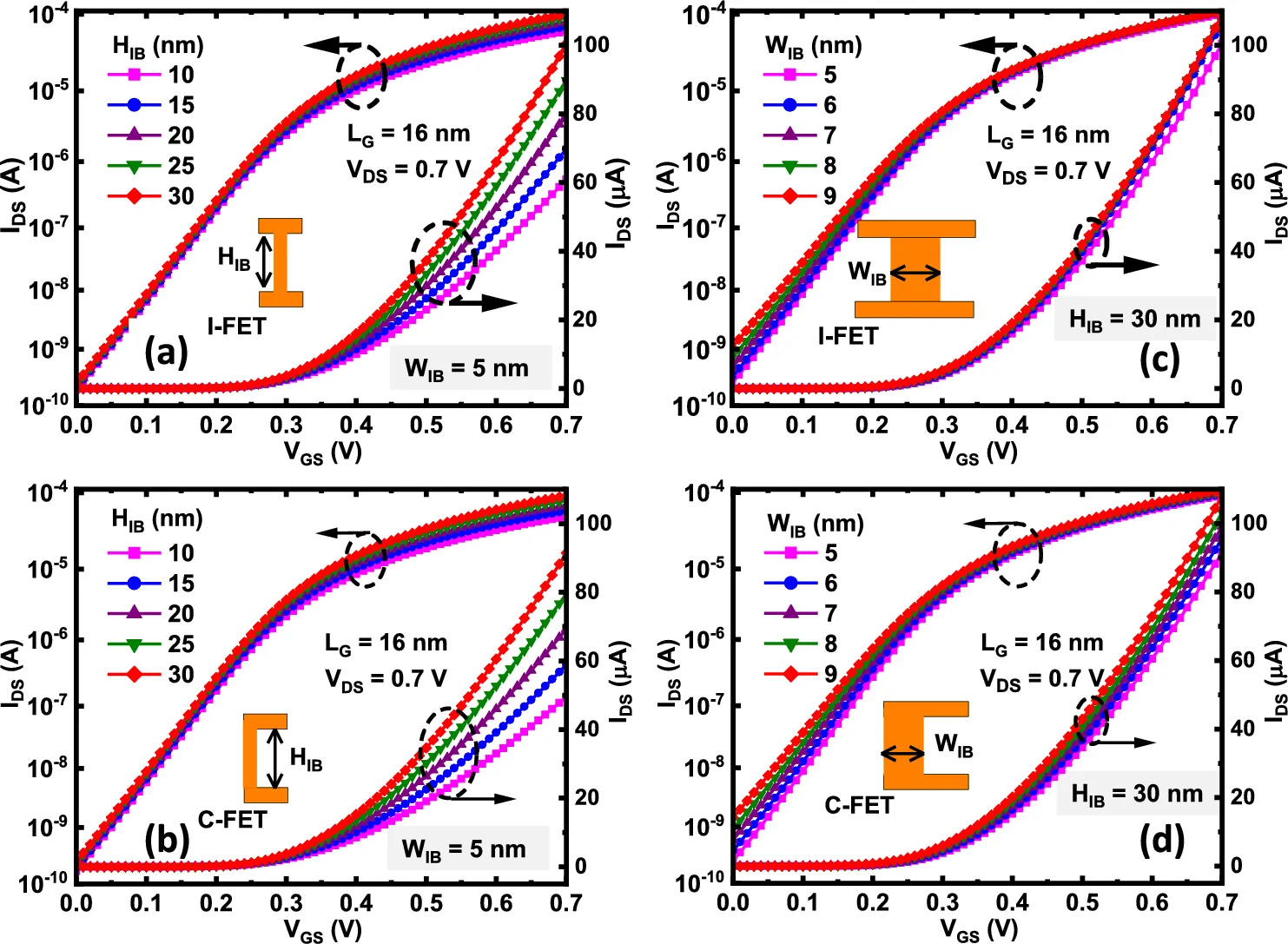
(**a**)–(**d**) $\mathrm {I_{DS}}$–$\mathrm {V_{GS}}$ characteristics of I-FET and C-FET while varying $\mathrm {H_{IB}}$ and $\mathrm {W_{IB}}$, respectively. Panels illustrate the relative sensitivity of drive current to vertical ($\mathrm {H_{IB}}$) versus lateral ($\mathrm {W_{IB}}$) IB scaling.

**Fig. 8:**
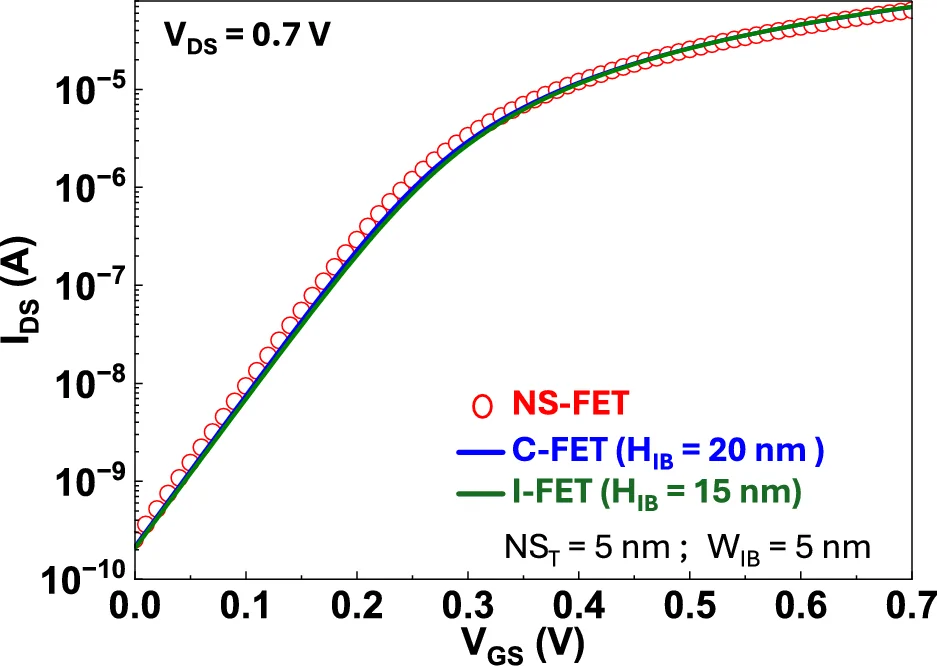
Comparison of the transfer characteristics of NS-FET, C-FET, and I-FET under reduced inter-bridge height ($H_{IB}$) conditions approaching nanosheet dimensions. As $H_{IB}$ decreases, the performance enhancement of C-FET and I-FET gradually reduces and converges toward conventional NS-FET behavior. The I-FET maintains performance comparable to NS-FET at relatively smaller $H_{IB}$ values than the C-FET due to its more efficient electrostatic coupling and centrally distributed inter-bridge conduction path.

**Fig. 9:**
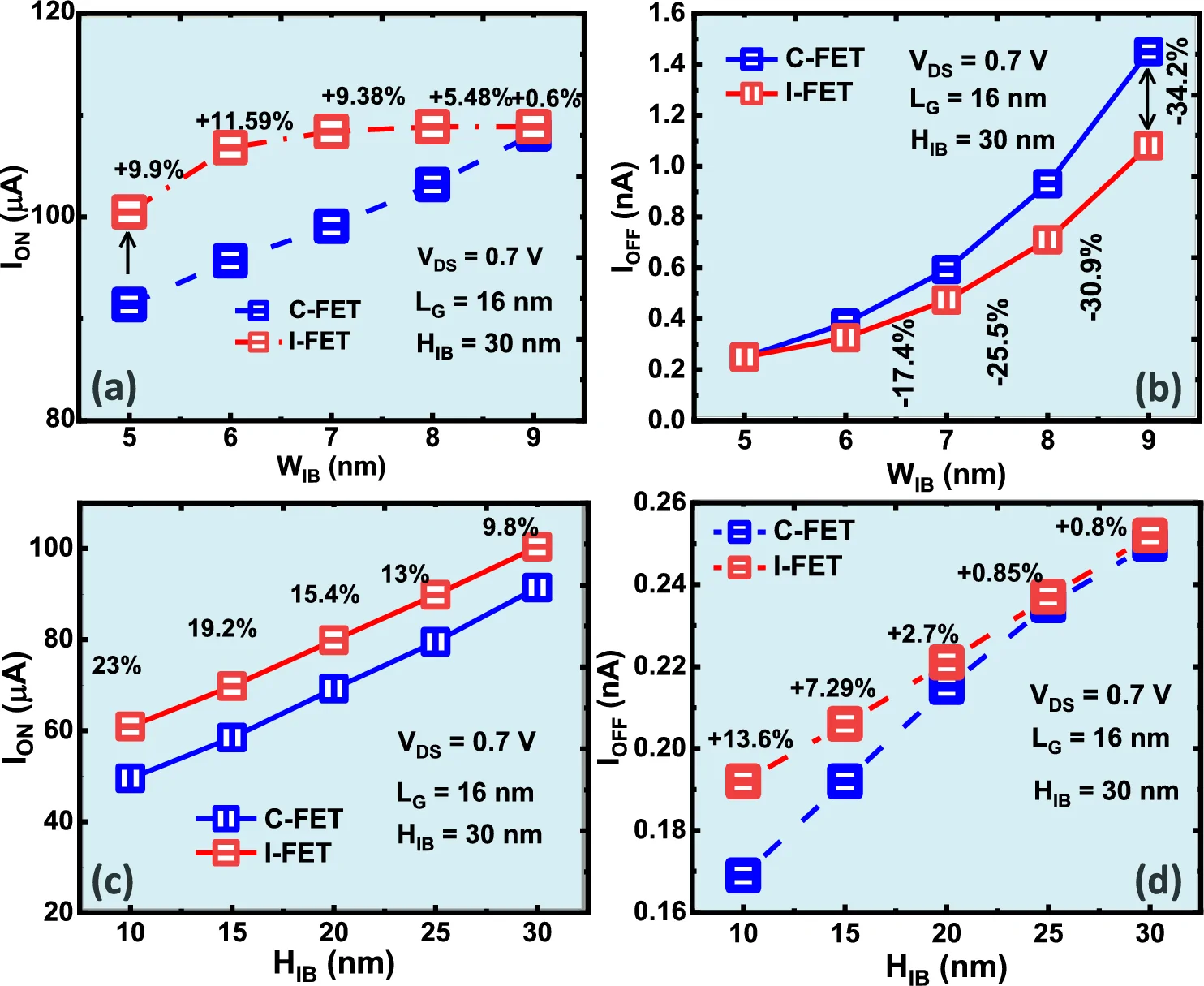
$\mathrm {I_{ON}}$ and $\mathrm {I_{OFF}}$ behavior of C-FET and I-FET for varied (**a**), (**b**) $\mathrm {W_{IB}}$ and (c),(d) $\mathrm {H_{IB}}$, respectively. Results show $\mathrm {H_{IB}}$ has a stronger, nearly linear effect on $\mathrm {I_{ON}}$ while $\mathrm {W_{IB}}$ produces only minor changes.

### Scaling impact of IB and $L_G$

Figure 6 illustrates the electric-field distribution for NS-FET, C-FET, and I-FET structures. The I-FET exhibits stronger and more uniformly distributed fields due to the additional vertical IB channel, resulting in improved carrier transport and higher $\mathrm {I_{DS}}$. Although stronger fields can potentially increase DIBL at high $\mathrm {V_{DS}}$, this concern is reduced in advanced nodes where lower supply voltages are used.

The effect of IB scaling is analyzed by independently varying the IB height ($\mathrm {H_{IB}}$) and width ($\mathrm {W_{IB}}$), as shown in Fig. 7. The selected IB height range (10–30 nm) spans values comparable to the nanosheet thickness and is used to systematically evaluate the performance trends of the proposed architectures. Increasing $\mathrm {H_{IB}}$ enhances $\mathrm {I_{DS}}$ for both C-FET and I-FET due to increased vertical conduction area and improved $\mathrm {W_{eff}}$. In contrast, scaling $\mathrm {W_{IB}}$ produces only a modest change in $\mathrm {I_{DS}}$ because lateral field contributions are inherently weaker than vertical gate-induced fields. In all cases, the I-FET maintains superior drive current due to its hybrid conduction paths. The observed performance trends remain consistent across the entire $H_{IB}$ range studied.

To further examine the fairness of comparison under reduced inter-bridge dimensions, Fig. 8 compares the transfer characteristics of NS-FET, C-FET, and I-FET for smaller $H_{IB}$ values approaching dimensions comparable to the nanosheet thickness. As $H_{IB}$ decreases, the performance enhancement of both C-FET and I-FET progressively reduces and gradually converges toward conventional NS-FET behavior. This trend is physically expected because reducing $H_{IB}$ decreases the additional effective conduction contribution introduced by the inter-bridge region, thereby reducing the effective width enhancement over the NS-FET structure.

Interestingly, the I-FET maintains comparable drive characteristics at relatively smaller $H_{IB}$ values compared to the C-FET. This behavior is attributed to the more symmetric electrostatic coupling and centrally distributed inter-bridge conduction path in the I-FET structure, which enables more efficient carrier transport between stacked nanosheets. In contrast, the C-FET relies more strongly on sidewall-connected conduction paths, requiring relatively larger $H_{IB}$ values to achieve similar enhancement. This interpretation is further supported by the electric-field contours shown in Fig. 6, where the I-FET exhibits stronger and more uniformly distributed electric fields across the inter-bridge region.

Figure 9 summarizes the impact of IB scaling on $\mathrm {I_{ON}}$ and $\mathrm {I_{OFF}}$. Increasing $\mathrm {W_{IB}}$ results in only small improvements in $\mathrm {I_{ON}}$, whereas scaling $\mathrm {H_{IB}}$ leads to a near-linear increase in $\mathrm {I_{ON}}$ for both devices. The I-FET consistently outperforms the C-FET, achieving up to an 11.6% improvement in $\mathrm {I_{ON}}$ across the evaluated range. Although $\mathrm {I_{OFF}}$ slightly increases with larger $\mathrm {H_{IB}}$, both devices maintain acceptable leakage levels, converging when their $\mathrm {W_{eff}}$ becomes comparable.

**Fig. 10:**
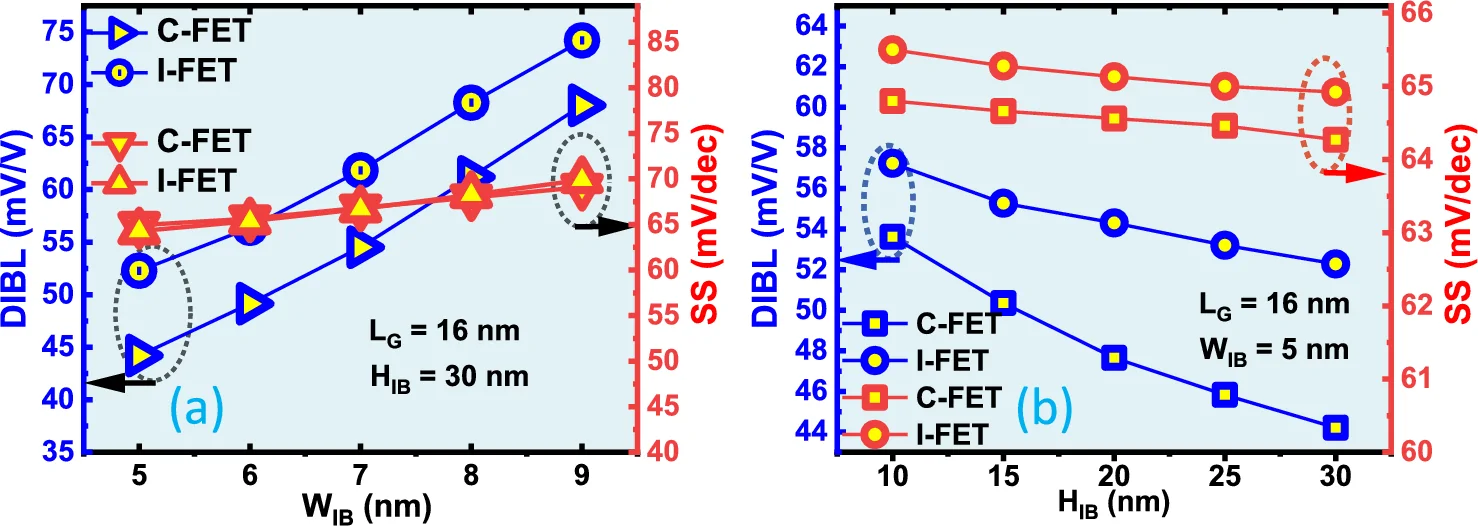
DIBL and SS of C-FET and I-FET at scaled (**a**) $\mathrm {W_{IB}}$ and (**b**) $\mathrm {H_{IB}}$, respectively. C-FET generally shows slightly better DIBL for increasing $\mathrm {W_{IB}}$, while increasing $\mathrm {H_{IB}}$ improves electrostatic control for both devices.

The DIBL and SS characteristics under IB scaling are presented in Fig. 10. For increasing $\mathrm {W_{IB}}$, both devices show a linear rise in DIBL due to weakened lateral electrostatic control, with C-FET exhibiting slightly better suppression than I-FET. In contrast, increasing $\mathrm {H_{IB}}$ improves electrostatic control and reduces DIBL, reflecting the dominance of the vertical gate field. SS values remain relatively stable across all conditions. Overall, minimizing $\mathrm {W_{IB}}$ while maximizing $\mathrm {H_{IB}}$ yields the best trade-off between drivability and short-channel control, with the I-FET offering the highest $\mathrm {I_{ON}}$ performance among the evaluated structures.

**Fig. 11:**
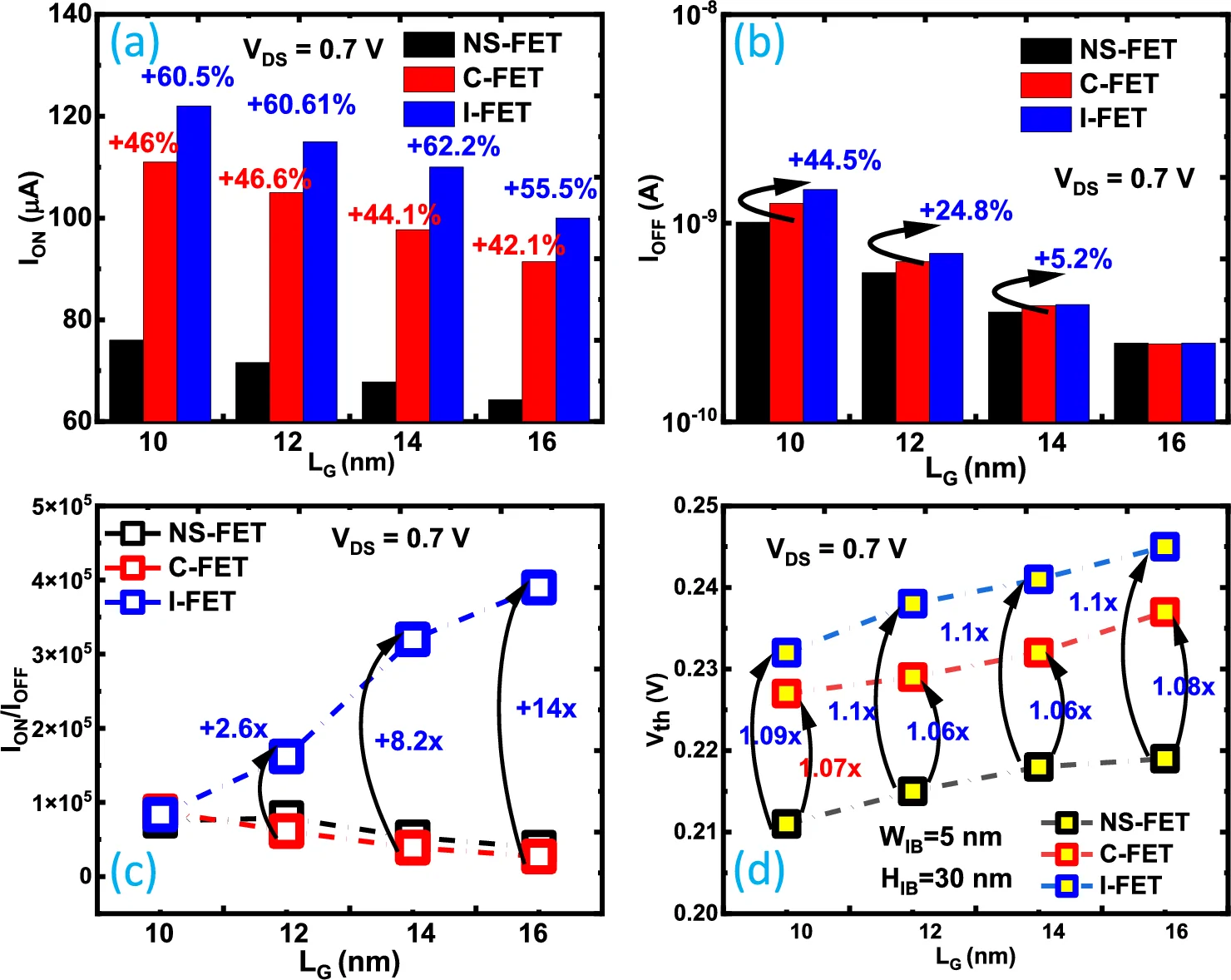
(**a**) $\mathrm {I_{ON}}$, (**b**) $\mathrm {I_{OFF}}$, (**c**) $\mathrm {I_{ON}}/\mathrm {I_{OFF}}$, and (**d**) $\mathrm {V_{th}}$ of NS-, C-, and I-FETs as a function of $\mathrm {L_G}$. Gate-length scaling increases $\mathrm {I_{ON}}$ for all devices, with the I-FET showing the largest relative gain while maintaining acceptable $\mathrm {I_{OFF}}$ and $\mathrm {V_{th}}$ behavior.

Finally, Fig. 11 presents the impact of $L_G$ scaling. As $L_G$ decreases from 16 nm to 10 nm, all devices show an increase in $\mathrm {I_{ON}}$, with the I-FET demonstrating the largest enhancement–up to 60.5% over NS-FET. While $\mathrm {I_{OFF}}$ increases slightly at shorter $L_G$, the $\mathrm {I_{ON}}/\mathrm {I_{OFF}}$ ratio for I-FET remains significantly higher than for NS-FET and C-FET–exceeding them by nearly an order of magnitude at the shortest gate length. The threshold voltage ($V_{\textrm{th}}$) of I-FET also scales favorably with reduced gate length, whereas the C-FET exhibits mild quantum confinement effects for $L_G \le 10$ nm. This highlights the robustness of the I-FET architecture for aggressive gate scaling.

In addition to the scaling analysis, it is important to consider practical aspects such as variability, reliability, and drain tunneling. Due to the gate-all-around electrostatics, the proposed C-FET and I-FET are expected to exhibit variability trends comparable to conventional nanosheet FETs, although the inter-bridge (IB) region may introduce additional sensitivity to geometric variations. From a reliability perspective, no new degradation mechanisms are anticipated beyond those in standard NS-FETs, as the devices operate under similar biasing and material conditions. Furthermore, the impact of drain-induced tunneling is inherently accounted for through the use of matched $I_{\textrm{OFF}}$ conditions, ensuring a fair and consistent comparison of device performance.

**Fig. 12:**
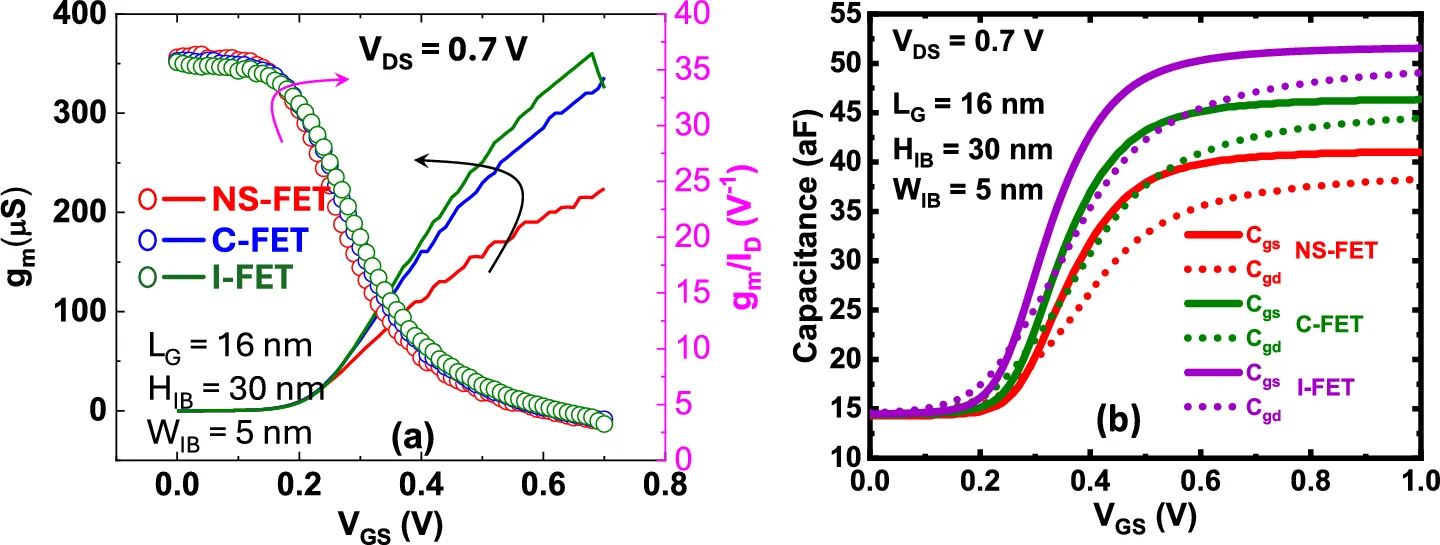
(**a**) Transconductance ($g_m$) and transconductance efficiency ($g_m/I_D$), and (**b**) $C_{gs}$ and $C_{gd}$ capacitances of NS-FET, C-FET, and I-FET, respectively.

**Fig. 13:**
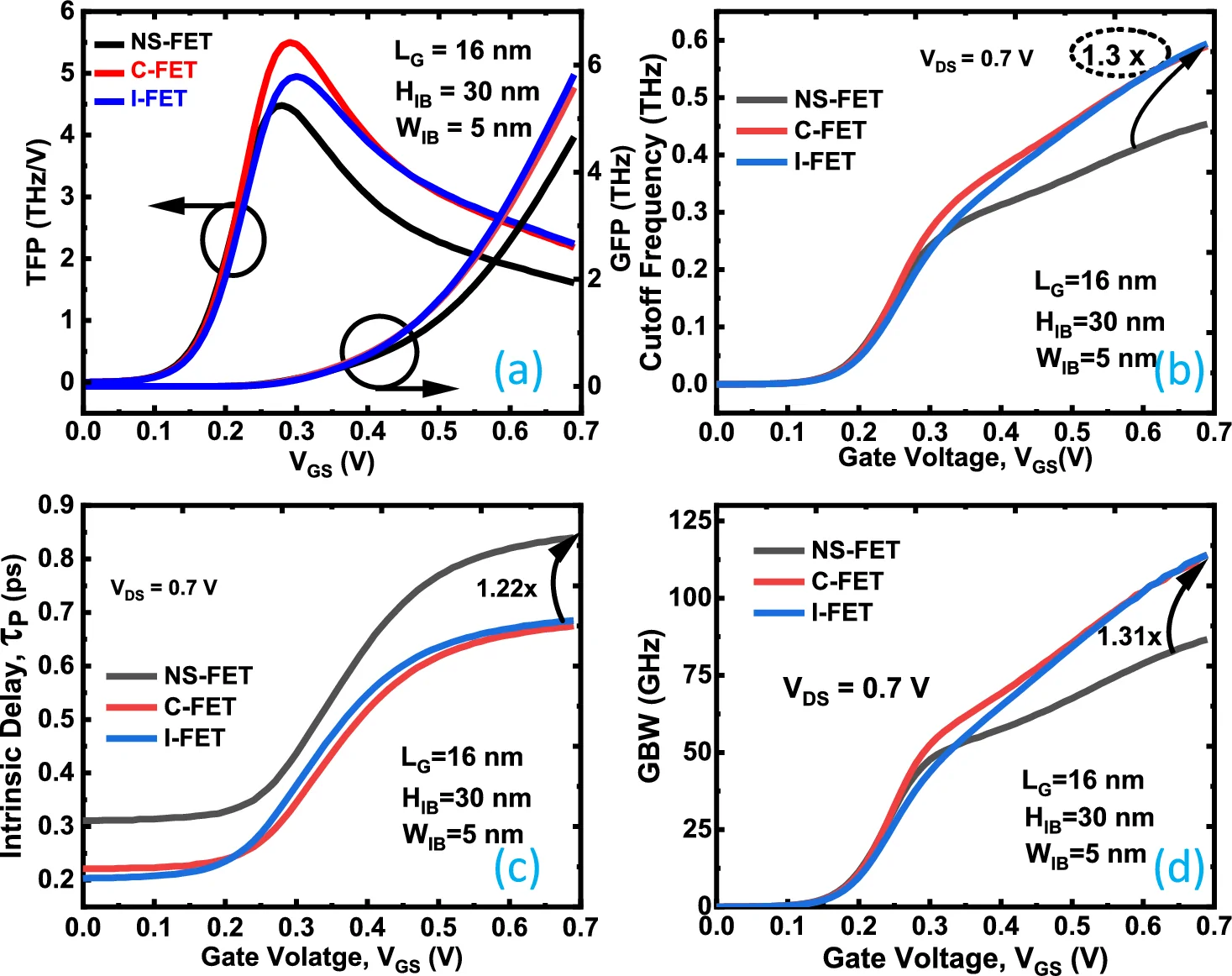
RF figures of merit: (**a**) TFP, (**b**) $f_{T}$, (**c**) $\tau _{p}$, and (**d**) GBW for NS-FET, C-FET, and I-FET.

**Fig. 14:**
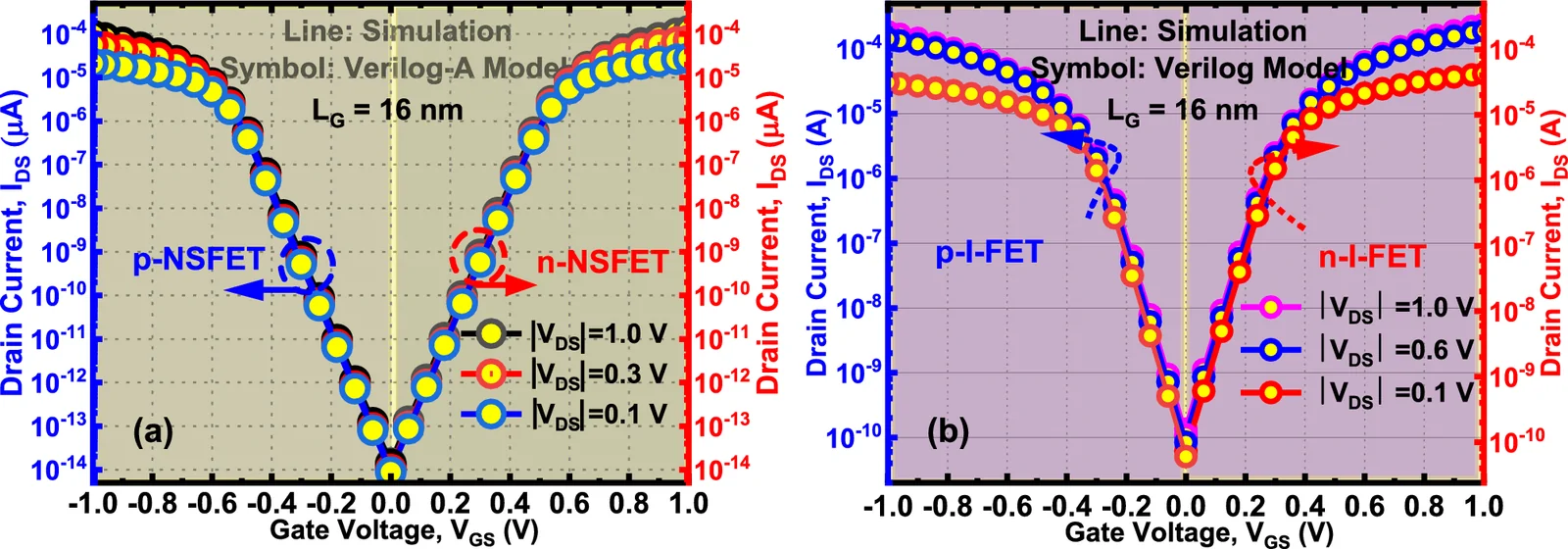
(**a**) Calibration of TCAD and lookup-table–based Verilog-A models for NS-FET and (**b**) I-FET by comparing $\mathrm {I_{DS}}$–$\mathrm {V_{GS}}$ characteristics at multiple $\mathrm {V_{DS}}$ values.

**Fig. 15:**
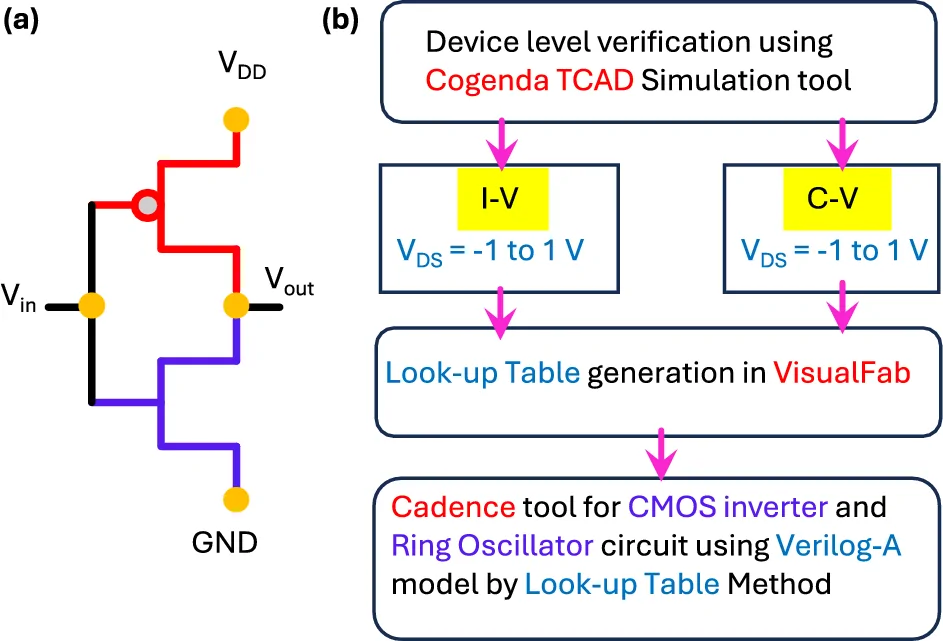
(**a**) CMOS inverter circuit implemented using NS-FET, C-FET, and I-FET devices. (**b**) Device-to-circuit simulation flow from TCAD extraction to Verilog-A modeling and Cadence implementation.

**Fig. 16:**
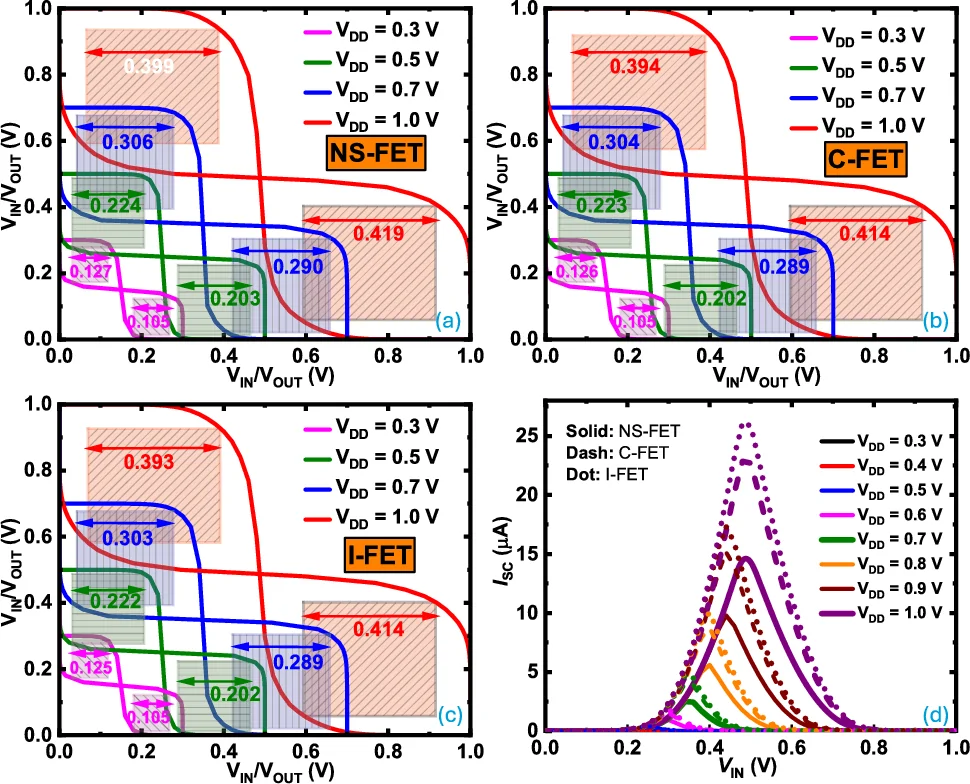
(**a**–**c**) CMOS inverter SNM butterfly curves for NS-FET, C-FET, and I-FET at various $\mathrm {V_{DD}}$; (**d**) switching current characteristics showing higher $\mathrm {I_{SC}}$ for I-FET.

### Analog and RF performance

This subsection evaluates the analog and RF performance of NS-FET, C-FET, and I-FET in terms of transconductance ($g_{m}$), transconductance efficiency ($g_m/I_D$), device capacitances, and key RF figures of merit. Figure 12a compares the $g_{m}$ characteristics of the three architectures. As $V_{GS}$ increases, all devices exhibit a monotonic rise in $g_{m}$ due to enhanced inversion charge, with the I-FET achieving the highest peak value of 350 *μ*S. This improvement is directly attributed to its enlarged effective conduction width enabled by the IB channel. The enhanced $g_{m}$ of I-FET translates to a higher unity-gain cutoff frequency $f_{T}$ and improved gain capability, making it suitable for both high-performance and RF applications.

The $g_m/I_D$ curves in Fig. 12a show similar trends for all devices–higher values at low $V_{GS}$ followed by a gradual decline, reflecting reduced transconductance efficiency in strong inversion. The $g_m/I_D$ characteristics were extracted using the logarithmic derivative relation $g_m/I_D = d(\ln I_D)/dV_{GS}$ to ensure numerical stability in the low-current regime. The peak $g_m/I_D$ values observed in the weak inversion region remain within the expected physical limit governed by the subthreshold swing at room temperature, while the moderate inversion region exhibits the most relevant operating range for analog/RF applications.

The capacitance components extracted for the three devices are shown in Fig. 12b. The total gate capacitance $C_{gg}$ is computed as the sum of $C_{gs}$ and $C_{gd}$, where $C_{gs}$ is primarily governed by inversion charge injection near the source, while $C_{gd}$ is influenced by depletion and Miller coupling at the drain-side junction. Among the three devices, the I-FET exhibits the highest $C_{gs}$ and $C_{gd}$ values due to its multi-surface channel geometry with additional corners and intersections. Nevertheless, all capacitance values remain within the attofarad (aF) range, ensuring compatibility with RF requirements and preventing excessive capacitive loading.

Beyond small-signal transconductance, additional RF metrics–namely the transconductance frequency product (TFP), gain frequency product (GFP), intrinsic delay ($\tau _{p}$), and gain–bandwidth product (GBW)–are analyzed in Fig. 13. TFP, which characterizes the trade-off between transconductance and switching speed, is found to be higher for C-FET in the mid-$V_{GS}$ region, while I-FET shows noticeable improvement at high $V_{GS}$ due to its steeper $g_{m}$ roll-up. The cutoff frequency $f_{T}$, shown in Fig. 13b, increases for both C-FET and I-FET relative to NS-FET, primarily due to their larger $g_{m}$ values. A maximum improvement of approximately *1.3×* over NS-FET is observed, indicating that the channel-engineered devices are better suited for nanoscale RF circuits. These high $f_{T}$ values also confirm that external fringing or overlap capacitances do not dominate device behavior.

The intrinsic delay $\tau _{p}$, plotted in Fig. 13c, decreases for both C-FET and I-FET compared to NS-FET, owing to their lower $C_{gg}$–to–$g_{m}$ ratio. Reduced delay directly benefits high-speed digital and mixed-signal applications. Finally, the gain–bandwidth product (GBW) shown in Fig. 13d demonstrates that both C-FET and I-FET provide superior GBW compared with NS-FET. Although the IB-based structures exhibit slightly higher $C_{gd}$, their substantially larger $g_{m}$ compensates for this, enabling higher achievable gain across a wide frequency range.

Overall, the analog and RF analysis confirms that C-FET and I-FET significantly outperform the conventional NS-FET in transconductance, cutoff frequency, delay, and bandwidth metrics. Among them, I-FET delivers the strongest overall performance due to its hybrid lateral–vertical conduction path, making it a promising candidate for future high-speed and RF-oriented technology nodes.

**Fig. 17:**
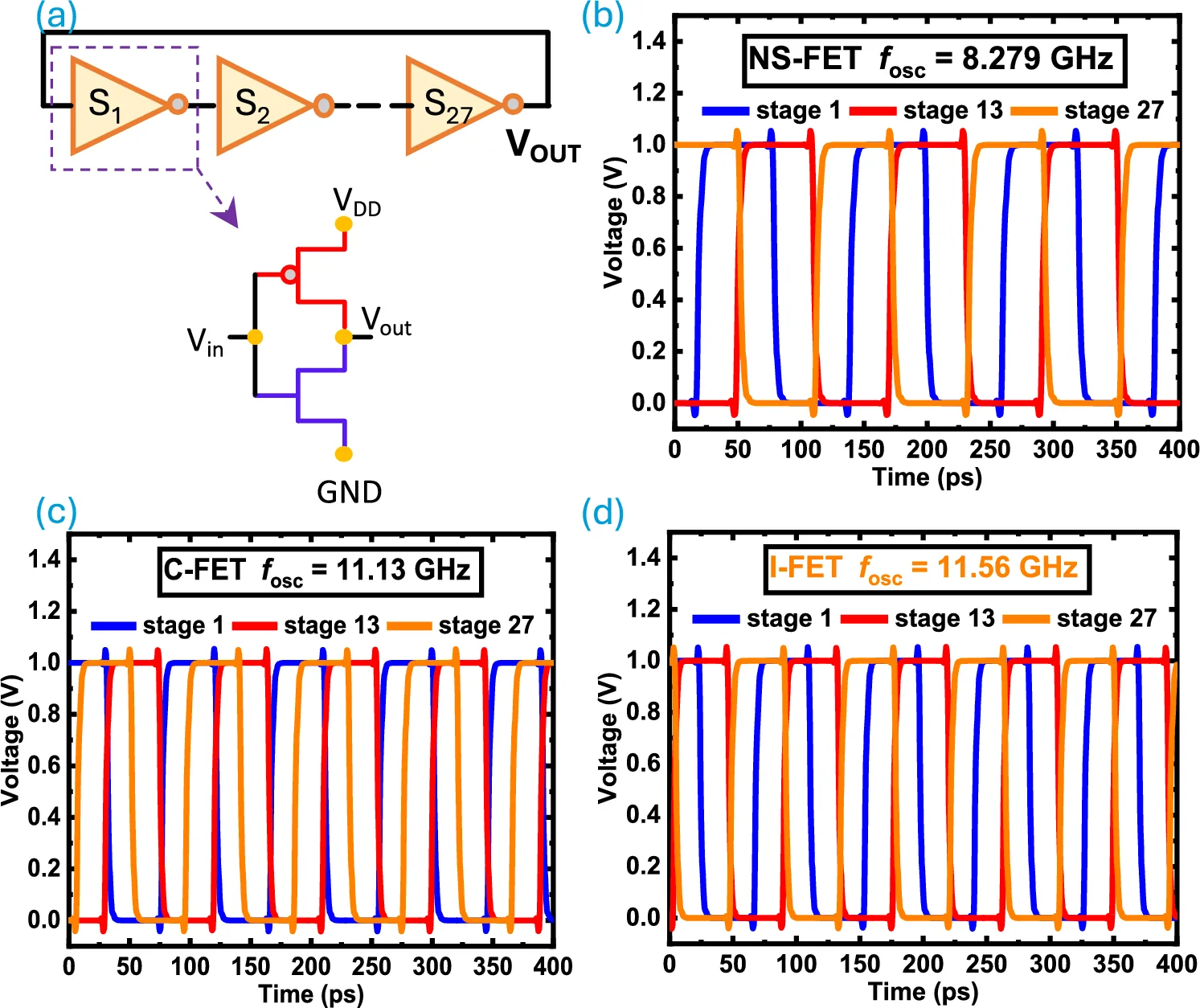
27-stage ring oscillator and corresponding output waveforms for **(a)** CMOS based ring oscillator circuit (**b**) NS-FET, (**c**) C-FET, and (**d**) I-FET implementations. Inset shows extracted $f_{\textrm{OSC}}$.

**Fig. 18:**
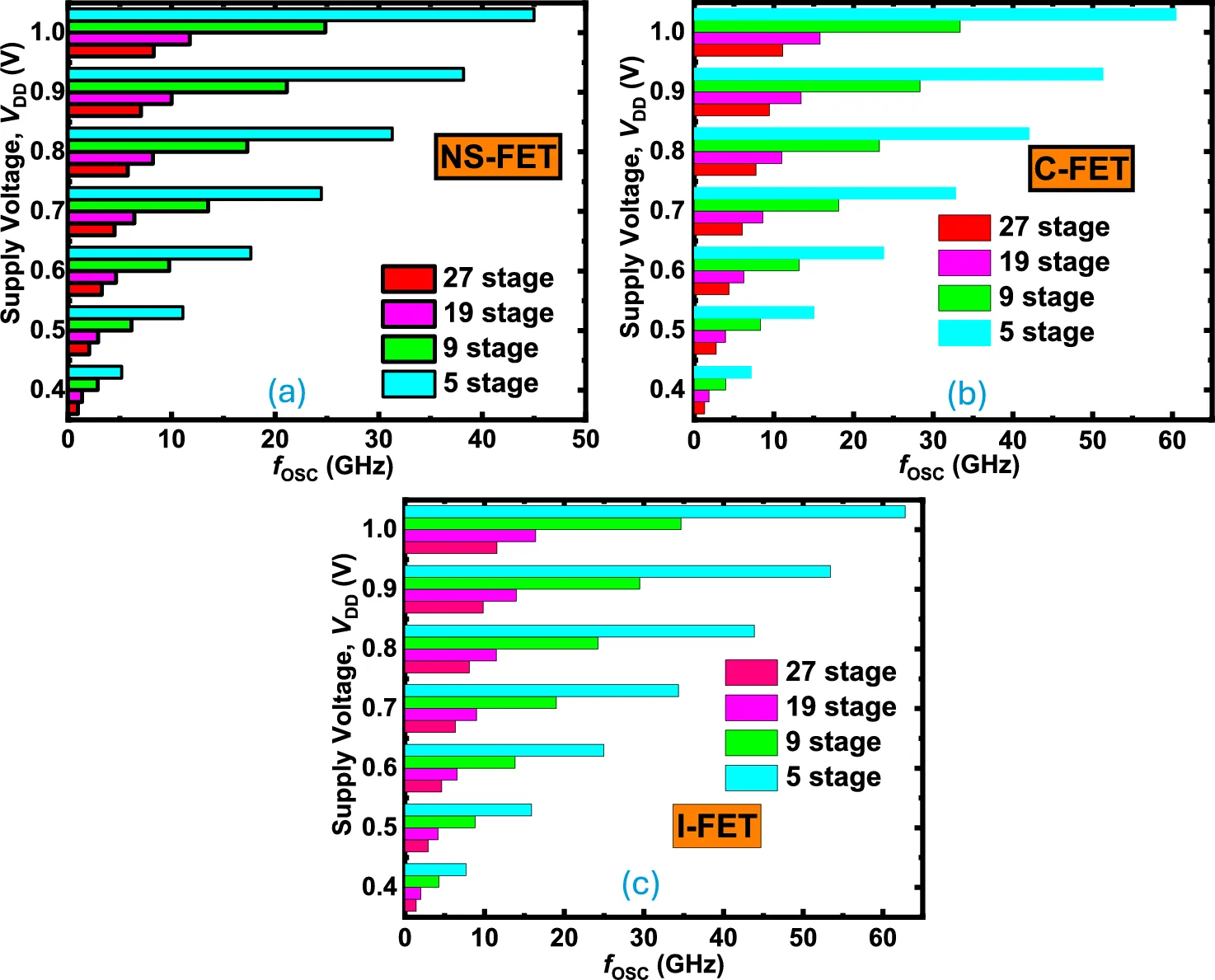
$f_{\textrm{OSC}}$ for N-stage ring oscillators implemented using (**a**) NS-FET, (**b**) C-FET, and (**c**) I-FET devices across different $V_{\textrm{DD}}$.

### Circuit-level analysis of NS-FET, C-FET, and I-FET inverter

The circuit-level behavior of the proposed NS-FET, C-FET, and I-FET devices is evaluated using the Cadence simulation environment. Device-level characteristics such as $\mathrm {I_{DS}}$–$\mathrm {V_{GS}}$, $\mathrm {I_{DS}}$–$\mathrm {V_{DS}}$, and C–V data are first extracted from 3D TCAD simulations. These characteristics are converted into a multi-dimensional lookup-table and embedded within a Verilog-A compact model, enabling seamless device-to-circuit integration. The accuracy of the Verilog-A interface is validated by comparing the lookup-table based Cadence output with TCAD results at multiple $\mathrm {V_{DS}}$ biases, as shown in Fig. 14.

Figure 15a shows the CMOS inverter schematic used for circuit evaluation, where NS-FET, C-FET, and I-FET serve as both PMOS and NMOS transistors for a 3 nm technology-node implementation. The overall simulation flow–from TCAD extraction to Verilog-A model generation and Cadence circuit execution–is illustrated in Fig. 15b. The voltage transfer characteristics (VTC) for different $\mathrm {V_{DD}}$ values are shown in Fig. 14b, where C-FET and I-FET inverters exhibit steeper transitions due to their higher $\mathrm {I_{ON}}$ and improved transconductance.

Figure 16a–c present the butterfly curves of NS-FET-, C-FET-, and I-FET-based CMOS inverters for $\mathrm {V_{DD}}$ values ranging from 0.3 V to 1.0 V. The signal noise margin (SNM) is extracted using the largest inscribed square method. At $\mathrm {V_{DD}} = 1$ V, the NS-FET achieves the highest noise margins (NMH = 0.399 V and NML = 0.419 V), followed closely by C-FET and I-FET. The slightly reduced SNM in C-FET and I-FET inverters is attributed to delayed saturation onset due to the inter-bridge geometry. Figure 16d shows the switching current ($I_{\textrm{SC}}$), where the I-FET delivers the highest peak current, consistent with its superior $\mathrm {I_{ON}}$.

The high-frequency performance is assessed using ring oscillator (RO) circuits of varying stage counts. Figure 17 shows the output oscillations of a 27-stage RO at $\mathrm {V_{DD}} = 1$ V. The oscillation frequencies ($f_{\textrm{OSC}}$) are 8.279 GHz, 11.13 GHz, and 11.56 GHz for NS-FET, C-FET, and I-FET implementations, respectively. The reduced propagation delay in I-FET-based inverters directly contributes to its highest $f_{\textrm{OSC}}$. Figure 18 further compares $f_{\textrm{OSC}}$ across different stage counts (5-, 9-, 19-, and 27-stage ROs) and supply voltages ranging from 0.4 V to 1.0 V. In all cases, I-FET maintains the highest oscillation frequency, making it the most favorable candidate for high-speed and RF-oriented circuits.

## Conclusions

This work presented a comprehensive device-to-circuit evaluation of channel-engineered GAA architectures–C-FET and I-FET–benchmarked against a conventional NS-FET for sub-3 nm technology nodes. The introduction of inter-bridge (IB) structures significantly enhanced the effective conduction width, enabling notable improvements in $I_{\textrm{ON}}$, $I_{\textrm{ON}}/I_{\textrm{OFF}}$, and analog/RF metrics. Among the devices studied, the I-FET demonstrated the highest drive current, superior transconductance, and improved cutoff frequency owing to its combined lateral and vertical channel pathways. While both C-FET and I-FET exhibited slightly higher SS and DIBL due to limited gate controllability in the IB region, these values remained within acceptable limits for future CMOS nodes.

Circuit-level validation using Verilog-A models in Cadence further confirmed the benefits of channel-engineered designs. The I-FET demonstrated the fastest switching behavior, higher inverter switching currents, and the largest ring-oscillator frequencies across all supply voltages and RO stages examined. The C-FET also outperformed the NS-FET in most circuit metrics, highlighting the overall advantage of IB-assisted architectures. These results collectively show that C-FET and especially I-FET devices offer strong potential for high-performance and RF applications, establishing them as promising candidates for continued CMOS scaling beyond advanced nanosheet technology. Future work will include variability-aware analysis, considering factors such as line-edge roughness and work-function variation, to assess the robustness of the proposed architectures under process-induced variations.

## Data Availability

The data that support the findings of this study are available from the corresponding author upon reasonable request.
